# Bibliography

**Published:** 1891-04

**Authors:** 


### BIBLIOGRAPHY.

A Dictionary of Dental Science and such Words and Phrases of the Collateral Sciences as pertain to the Art and Practice of Dentistry. By Chapin A. Harris, M.D., D.D.S. Fifth edition, carefully revised and enlarged by Ferdinand J. S. Gorgas, M.D., D.D.S. P. Blakiston, Son & Co., Philadelphia, 1891.

This dictionary has been so long before the dental profession, having been prepared by the late Professor Harris in 1849, that any extended review seems almost out of place. The changes and additions made in this edition since the last, in 1877, almost give dental readers a new work of reference, and tend "to make it a more purely dental work than ever before." That this has been accomplished must be conceded, and an examination shows generally careful revision by one competent to effect the necessary changes. "Many hundred words and definitions" have been added, bringing it up to the present period, with the additions made to the nomenclature of dentistry as well as that of collateral sciences.

It is, therefore, with no spirit of criticism that attention is called to several omissions observed in glancing over the pages.

Under the word *Pyorrhœa* is given *Pyorrhœa Alveolaris*, and reference is made to *Alveolar Pyorrhœa.* On turning to the proper page this latter word is not to be found. Indeed, this pathological condition is not properly defined anywhere, the nearest approach being under *Phagedenic Pericementitis ;* but it is not there satisfactorily explained.

The description of *Rubber Dam*, or *Barnum's Coffer Dam*, is full enough ; but does not do justice to Dr. Barnum. A dictionary of this kind is in one sense a history of the origin of things, and it will be referred to for information by the future collector of facts, and hence should not only be carefully prepared in its definitions, but should be exact in giving credit to original workers. This Professor Gorgas has failed to do in many instances, notably under *Bleaching Teeth*, where he quotes the entire process of the writer of this without the slightest credit.

The various definitions under *Gold* are exceptionally good.

For the dentist this is unquestionably the best dictionary for reference, and not only the best, but the only one extant where he will find all the technical terms used in his profession.

A Compend of Human Anatomy. By Samuel V. L. Potter, M.A., M.D. Fifth edition. One hundred and seventeen wood-engravings, numerous tables, and sixteen lithographic plates of the nerves and arteries. P. Blakiston, Son & Co., Philadelphia, 1890.

This book has shown by the numerous editions that it has been greatly appreciated by the class for whom it was originally intended, —viz., medical students preparing for the exercises of the quiz-room or for final examination. It is arranged for its special object with care and excellent judgment.

The present edition has an appendix of forty-three pages, containing a complete set of tables and plates of the arteries, the cranial and spinal nerves and plexuses, and the sympathetic nervous system.

ILLUSTRATED CATALOGUE. The Wilmington Dental Manufacturing Co., Philadelphia, 1890.

This comparatively young and energetic corporation presents to the dental profession this well-prepared catalogue. The preface gives a short history of the rise and progress of this company. It was incorporated in 1882. In the year 1886 a controlling interest was secured in the Welch Dental Company, of Philadelphia, and in 1889 the latter was merged into the Wilmington Dental Manufacturing Company, and in May, 1890, the interests were consolidated with the American Dental Manufacturing Company, of New York. This is certainly a remarkable development in the period named, eight years.

VERHANDLUNGEN DER DEUTSCHEN ODONTOLOGISCHEN GESELLSCHAFT. Berlin, 1891.

The proceedings of this society, under the leadership of Professor Busch and Professor Warnekros as president and secretary, are published regularly in pamphlet form. The papers show in many respects a more satisfactory statement of facts than is customary on this side of the ocean, and in the preparation of illustrations and exactness in following details there is left nothing to be desired.

